# Supplementary material for: Steering eco-evolutionary game dynamics with manifold control
Source: Proc Math Phys Eng Sci. 2020 Jan 8;476(2233):20190643. doi: 10.1098/rspa.2019.0643 (PMC7016546; doi:10.1098/rspa.2019.0643)
Supplement: Appendix A: Stability of boundary fixed points when the feedback control function f is in quadratic forms.;Appendix B: Constructive proof for the existence of control laws in the general framework. [file rspa20190643supp1.zip › ESM/SupplementaryMaterials.pdf]

Appendix A: Stability of boundary fixed points when the feedback control function f is in quadratic forms.

Here we discuss the stability of all possible fixed points on the boundary (not include the intersection of equilibrium curve and the boundary, which is considered as a part of equilibrium curve) when the feedback control f is in quadratic forms, using parameters shown in **Fig. 2** in the main text. The co-evolutionary model can be written as

$$\begin{cases} \dot{x} = x(1-x)\left(P_c - P_d\right) \\ \dot{r_c} = \epsilon(r_c - \alpha)(\beta - r_c)(P_c - P_d)(a_1 - (\theta_1 P_c - P_d)) \end{cases} \tag{1}$$

in which

$$\begin{aligned} P_c &= \frac{1+Sx}{S+1} r_c - 1 \\ P_d &= \frac{Sx}{S+1} r_d \end{aligned} \tag{2}$$

Applying Eq. **??** as well as the fixed parameters $\alpha = 1.5$, $\beta = 3.5$, $S = 3$, $r_d = 1.5$, $\epsilon = 2$, $\theta_1 = 2$ into Eq. **??**, we have

$$\begin{cases} \dot{x} = x(1-x)(\frac{1+3x}{4} r_c - 1 - \frac{4.5}{4} x) \\ \dot{r_c} = 2(r_c - 1.5)(3.5 - r_c)(\frac{1+3x}{4} r_c - 1 - \frac{4.5}{4} x)(a_1 - (2(\frac{1+3x}{4} r_c - 1) - \frac{4.5}{4} x)) \end{cases} \tag{3}$$

Denote J as the Jacobian matrix of the system. We have five boundary fixed points in total:

(1) $x = 0, r_c = 1.5$

$$J(x=0, r_c=1.5) = \begin{bmatrix} -\frac{5}{8} & 0 \\ 0 & -\frac{5a_1}{2} - \frac{25}{8} \end{bmatrix}$$

The eigenvalues of the Jacobian matrix are $\lambda_1 = -\frac{5}{8} < 0$ and $\lambda_2 = -\frac{5a_1}{2} - \frac{25}{8} < 0$, since $a_1 > 0$ in our framework. Therefore the fixed point $(x = 0, r_c = 1.5)$ is always stable.

(2)$x = 0, r_c = 3.5$.

$$J(x=0, r_c=3.5) = \begin{bmatrix} -\frac{1}{8} & 0 \\ 0 & \frac{a_1}{2} + \frac{1}{8} \end{bmatrix}$$

The eigenvalues of the Jacobian matrix are $\lambda_1 = -\frac{1}{8} < 0$ and $\lambda_2 = \frac{a_1}{2} + \frac{1}{8} > 0$. Therefore the fixed point $(x = 0, r_c = 3.5)$ is always unstable.

(3) $x = 1, r_c = 1.5$.

$$J(x=1, r_c=1.5) = \begin{bmatrix} \frac{5}{8} & 0 \\ 0 & -\frac{5a_1}{2} - \frac{5}{16} \end{bmatrix}$$

The eigenvalues of the Jacobian matrix are $\lambda_1 = \frac{5}{8} > 0$ and $\lambda_2 = -\frac{5a_1}{2} - \frac{5}{16} < 0$. Therefore the fixed point $(x = 1, r_c = 1.5)$ is always unstable.

(4) $x = 1, r_c = 3.5$.

$$J(x=1, r_c=3.5) = \begin{bmatrix} -\frac{11}{8} & 0 \\ 0 & \frac{341}{16} - \frac{11a_1}{2} \end{bmatrix}$$

The eigenvalues of the Jacobian matrix are $\lambda_1 = -\frac{11}{8} < 0$ and $\lambda_2 = \frac{341}{16} - \frac{11a_1}{2}$. In the main text, we change $a_1 = 2, 0.5, 0$ respectively. Under all these circumstances, $\lambda_2 > 0$. Therefore the fixed point $(x = 1, r_c = 3.5)$ is always unstable.

(5) $x = 1, r_c = \frac{25}{16} + \frac{a_1}{2}$, which is the intersection of the control curve $\theta_i P_c - P_d = a_i$ and the boundary $x = 1$.

$$J(x=1, r_c=\frac{25}{16} + \frac{a_1}{2}) = \begin{bmatrix} \frac{9}{16} - \frac{a_1}{2} & 0 \\ (\frac{a_1}{2} - \frac{9}{16})(\frac{a_1}{2} - \frac{31}{16})(a_1 + \frac{1}{8})(\frac{3a_1}{4} + \frac{39}{32}) & 2(\frac{a_1}{2} - \frac{9}{16})(\frac{a_1}{2} - \frac{31}{16})(a_1 + \frac{1}{8}) \end{bmatrix}$$

The eigenvalues of the Jacobian matrix are $\lambda_1 = \frac{9}{16} - \frac{a_1}{2}$ and $\lambda_2 = 2(\frac{a_1}{2} - \frac{9}{16})(\frac{a_1}{2} - \frac{31}{16})(a_1 + \frac{1}{8})$. Therefore, when $a_1 \in (\frac{9}{8}, \frac{31}{8})$, we have $\lambda_1 < 0$ and $\lambda_2 < 0$ at the same time, the fixed point $(x = 1, r_c = \frac{25}{16} + \frac{a_1}{2})$ is stable. When $a_1 \in [0, \frac{9}{8})$ or $a_1 > \frac{31}{8}$, however, the fixed point is unstable.

In conclusion, we analyze the stability of all five boundary fixed points in **Fig. 2** in the main text, among which $(x = 0, r_c = 1.5)$ is always stable and $(x = 0, r_c = 3.5)$, $(x = 1, r_c = 1.5)$, $(x = 1, r_c = 3.5)$ are always unstable, while the stability of $(x = 1, r_c = \frac{25}{16} + \frac{a_i}{2})$ depends on the parameter a_i.

Appendix B: Constructive proof for the existence of control laws in the general framework.

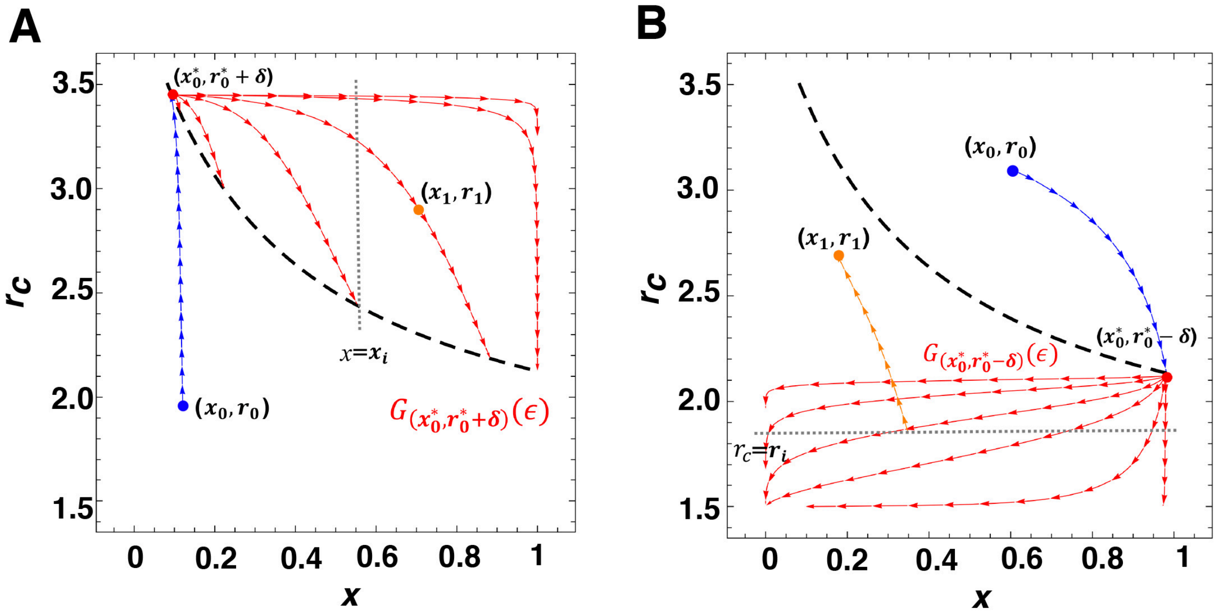

Figure S1: The existence of control laws for any desired final state (x_0, r_0) with a given initial state (x_1, r_1). (A) (x_0, r_0) is beneath the equilibrium curve while (x_1, r_1) is over the equilibrium curve. (B) (x_0, r_0) is over the equilibrium curve while (x_1, r_1) is beneath the equilibrium curve.

Here we provide a constructive proof for the existence of control laws when given a certain final state (x_1, r_1) with an initial state (x_0, r_0). For simplicity and without loss of generality, we fix the following parameters: $\alpha = 1.5, \beta = 3.5, S = 3, r_d = 1.5$ in our general framework.

(i) In **Fig. S1A**, we prove the existence of control laws when (x_0, r_0) is beneath the equilibrium curve while (x_1, r_1) is over the equilibrium curve. Under the fixed parameters, when $x_0 \le 1/12$, the initial fraction of cooperators is too small that the system will finally evolve into a mutual defection state in our framework. Assume $x_0 > 1/12$. (a) Firstly, we prove that we can control any given initial state evolving to the stable fixed point on the equilibrium curve that can arbitrarily approaches to the endpoint $(1/12, 3.5)$. When x_0 is close to $1/12$, we can simply use $f_1 = (P_c - P_d)((2P_c - P_d) - 1.2)(2 - (2P_c - P_d))$ with time-dependent switching control law. Let ϵ be sufficiently large, then (x_0, r_0) will finally stop at a stable fixed point (x_0^*, r_0^*) which can arbitrarily approaches to $(1/12, 3.5)$ on the equilibrium curve, like shown in the figure. When x_0 is relatively large, we may use state-dependent control laws in which $f_0 = (P_c - P_d)(2 - (2P_c - P_d))$ and change ϵ to control the initial state evolving to a desired new region where x_0 is close to $1/12$ in advance. Then similarly, we use f_1 and let ϵ be large enough to control the system evolving to the stable fixed point (x_0^*, r_0^*). (b) Then we prove that the trajectories begin from $(x_0^*, r_0^* + \delta)$ which is close to $(1/12, 3.5)$ can actually cover the whole region over the equilibrium curve. Here δ denotes a small disturbance on r_0^*. We present a possible control function $f_2 = (P_c - P_d)(0 - (4P_c - P_d))$. Define $G_{(x_0^*, r_0^*+\delta)}(\epsilon)$ as the set of trajectories that begin from $(x_0^*, r_0^* + \delta)$ with control function f_2 and relative feedback speed ϵ. $G_{(x_0^*, r_0^*+\delta)}(\epsilon)$ is a continuous function of t, x, r_c and ϵ. When ϵ is small enough, $G_{(x_0^*, r_0^*+\delta)}(\epsilon)$ finally stops at $(1, 2.125)$ which is the endpoint of the equilibrium curve. Meanwhile, when $\epsilon \to \infty$, the trajectory stops at a stable fixed point that infinitely approaches to (x_0^*, r_0^*). According to the continuity of ϵ, we derive that the trajectory set $G_{(x_0^*, r_0^*+\delta)}(\epsilon)$ can end at any fixed point ranging from (x_0^*, r_0^*) to $(1, 2.125)$. Further, given any $x = x_i$, when $\epsilon \to 0$, $G_{(x_0^*, r_0^*+\delta)}(0)$ goes through (x_i, r_0^*) while we already proved that there exists a ϵ_i that makes $G_{(x_0^*, r_0^*+\delta)}(\epsilon_i)$ stop at (x_i, r_i) on the equilibrium curve, in which $r_i = 1.5 + 2.5/(3x_i + 1)$. Again the continuity of ϵ results in traversal for all the points on $x = x_i$, $r_c \in [r_i, r_0^*]$ by $G_{(x_0^*, r_0^*+\delta)}(\epsilon)$. Therefore, we conclude that $G_{(x_0^*, r_0^*+\delta)}(\epsilon)$ can go through any point in the region $\{(x, r_c) : x \in (x_0^*, 1], r_c \in [1.5 + 2.5/(3x + 1), r_0^*]\}$. Let $(x_0^*, r_0^*) \to (1/12, 3.5)$, we come to the conclusion that the trajectories $G_{(x_0^*, r_0^*+\delta)}(\epsilon)$ cover the whole region over the equilibrium curve, which indicates the existence of control laws to reach to any desired (x_1, r_1) that is over the equilibrium curve.

(ii) In **Fig. S1B**, we prove the existence of control laws when (x_0, r_0) is over the equilibrium curve while (x_1, r_1) is beneath the equilibrium curve. (a) Firstly, we can control any given (x_0, r_0) evolves to a stable fixed point (x_0^*, r_0^*) that can arbitrarily approaches to $(1, 2.125)$ using time-dependent switching control laws with control function $f_1 = (P_c - P_d)(0 - (4P_c - P_d))$ and a proper relative feedback speed ϵ, as already proved in (i). (b) Define $G_{(x_0^*, r_0^*-\delta)}(\epsilon)$ as the set of trajectories that begin from $(x_0^*, r_0^* - \delta)$ with a certain control function f_2 and relative feedback speed ϵ. Let $f_2 = (P_c - P_d)(2 - (2P_c - P_d))$. Similarly we can prove that $G_{(x_0^*, r_0^*-\delta)}(\epsilon)$ goes through all the points in the region $\{(x, r_c) : x \in [0, x_0^*), r_c \in [1.5, r_0^* - \delta)\}$. Randomly choose a $r_i < r_0^*$, then we can reach to any point on $r = r_i$, as shown in the figure. Finally, for any final state (x_1, r_1) beneath the equilibrium curve that satisfies $r_1 \geq r_0^* - \delta$, there exists a unique trajectory which belongs to the phase space of the co-evolutionary system with control function $f_3 = (P_c - P_d)((2P_c - P_d) - 1.2)(2 - (2P_c - P_d))$ and a sufficiently large feedback speed ϵ, that goes through (x_1, r_1). Assume the intersection of this trajectory and $r = r_i$ is (x_i, r_i), then we have a possible control path from $(x_0^*, r_0^* - \delta)$ to (x_i, r_i) and finally reaches to (x_1, r_1). In summary, we can always find a group of control laws given any (x_0, r_0) that is over the equilibrium curve and (x_1, r_1) that is beneath the equilibrium curve.

Combining (i)(ii) which actually provide a possible control loop in phase space, we constructively prove the existence of control laws given any initial state (x_0, r_0) and final state (x_1, r_1), as long as the initial fraction of cooperators x_0 is not too small.
