# Supplementary figures and images for: Steering eco-evolutionary game dynamics with manifold control
Source: Proc Math Phys Eng Sci. 2020 Jan 8;476(2233):20190643. doi: 10.1098/rspa.2019.0643 (PMC7016546; doi:10.1098/rspa.2019.0643)

**A**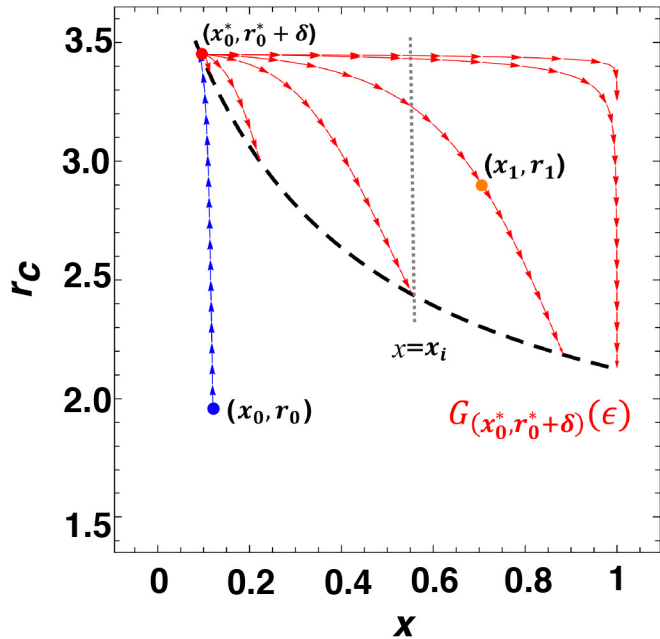**B**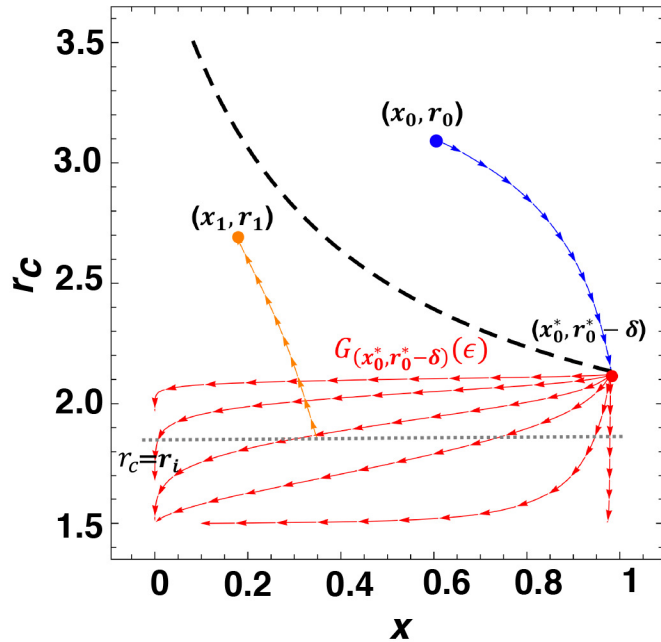

Supplement: Appendix A: Stability of boundary fixed points when the feedback control function f is in quadratic forms.;Appendix B: Constructive proof for the existence of control laws in the general framework. [file rspa20190643supp1.zip › ESM/SI_1-eps-converted-to.pdf]
